# Comprehensive MRI assessment reveals subtle brain findings in non-hospitalized post-COVID patients with cognitive impairment

**DOI:** 10.3389/fnins.2024.1435218

**Published:** 2024-09-10

**Authors:** Serena Fineschi, Markus Fahlström, David Fällmar, Sven Haller, Johan Wikström

**Affiliations:** ^1^Department of Public Health and Caring Sciences, Faculty of Medicine, Uppsala University, Uppsala, Sweden; ^2^Östhammar Health Care Centre, Östhammar, Sweden; ^3^Department of Surgical Sciences, Faculty of Medicine, Uppsala University, Uppsala, Sweden; ^4^Department of Radiology, Uppsala University Hospital, Uppsala, Sweden; ^5^Division of Radiodiagnostic and Interventional Radiology, Faculty of Medicine, University of Geneva, Geneva, Switzerland

**Keywords:** post-COVID, MRI, attention network, cognitive impairment, resting state fMRI, right middle frontal gyrus, right temporoparietal junction

## Abstract

**Background:**

Impaired cognitive ability is one of the most frequently reported neuropsychiatric symptoms in the post-COVID phase among patients. It is unclear whether this condition is related to structural or functional brain changes.

**Purpose:**

In this study, we present a multimodal magnetic resonance imaging study of 36 post-COVID patients and 36 individually matched controls who had a mild form of severe acute respiratory syndrome coronavirus 2 (SARS CoV-2) infection from March 2020 to February 2022. This study aimed to investigate structural and functional brain alterations and their correlation with post-COVID symptoms and neurocognitive functions.

**Materials and methods:**

The study protocol comprised an assessment of physical fatigue [Fatigue Severity Scale (FSS)], mental fatigue (Mental Fatigue Scale (MFS)], depression [Montgomery Asberg Depression Rating Scale (MADRS)], anxiety [Hospital Anxiety and Depression Scale (HAD)], post-COVID Symptoms Severity Score, and neurocognitive status [Repeatable Battery for the Assessment of Neuropsychological Status Update (RBANS)]. The magnetic resonance imaging protocol included morphological sequences, arterial spin labeling (ASL) and dynamic susceptibility contrast-enhanced (DSC) perfusion, diffusion tensor imaging (DTI), and resting-state functional magnetic resonance imaging (fMRI) sequences. Using these protocols, the assessments of macrostructural abnormalities, perfusion, gray matter density, white matter integrity, and brain connectivity were performed.

**Results:**

Post-COVID patients had higher levels of physical fatigue, mental fatigue, depression, and anxiety than controls and showed cognitive impairment in all the RBANS domains except in Visuospatial/Construction. The subjective mental fatigue correlated with objective impaired cognitive ability in the RBANS test, particularly in the Attention domain. There were no differences between patients and controls regarding macrostructural abnormalities, regional volumes, regional perfusion metrics, gray matter density, or DTI parameters. We observed a significant positive correlation between RBANS Total Scale Index score and gray matter volume in the right superior/middle-temporal gyrus (*p* < 0.05) and a significant negative correlation between the white matter integrity and post-COVID symptoms (*p* < 0.05) in the same area. The connectivity differences were observed between patients and controls in a few regions, including the right middle frontal gyrus, an important area of convergence of the dorsal and ventral attention networks. We also noted a positive correlation between post-COVID symptoms and increased connectivity in the right temporoparietal junction, which is part of the ventral attention system.

**Conclusion:**

In non-hospitalized subjects with post-COVID, we did not find any structural brain changes or changes in perfusion, compared to controls. However, we noted differences in connectivity within an important area for attention processes, which may be associated with post-COVID brain fog.

## Introduction

Post-COVID condition occurs in individuals with a history of probable or confirmed SARS CoV-2 infection, usually 3 months from the onset of COVID-19, with symptoms that last for at least 2 months and cannot be explained by an alternative diagnosis, according to Delphi consensus, 2021 (Soriano et al., 2022).

Impaired cognitive ability and physical fatigue are the most frequently reported neuropsychiatric symptoms in post-COVID followed by insomnia, depression, and anxiety (Badenoch et al., 2022).

Other common physical symptoms are dyspnea, postural orthostatic tachycardia syndrome (POTS), widespread muscular pain, dizziness, and headache. Physical and mental fatigue are often accompanied by exertional intolerance and post-exertional malaise. Self-reported word*-*finding difficulties are among the most frequent neurocognitive complaints, such as impaired memory and attention. Cognitive exhaustion can worsen after even very light physical or mental exertion and leads to social isolation and long-term sick leave. A subset of post-COVID patients also fulfills the diagnostic criteria for myalgic encephalomyelitis (ME) and chronic fatigue syndrome (CFS) (Bonilla et al., 2023).

Depression, sleep disturbance, and anxiety, which have been reported to appear at the same time as the other post-COVID symptoms and are unlikely to be a mere consequence of the impaired cognitive ability, may affect the patient's experience of fatigue and their perception of cognitive impairment (Jaltuszewska et al., 2023).

Many studies show that neuropsychiatric symptoms also occur after a mild COVID infection (Graham et al., 2021) and can persist up to 2 years (Fernández-de-Las-Peñas et al., 2022).

The SARS-CoV-2 virus affects the brain directly from the cribriform plate, which is situated close to the olfactory bulb, or follows a hematogenous route (Singh et al., 2023). At the histopathological level, the three most common brain abnormalities reported during the acute phase of infection are inflammation, hypoxia, and coagulation disorders (Lu et al., 2020). However, the mechanism underlying the pathophysiology of neurologic symptoms in the post-COVID phase, especially after a mild COVID infection, is still debated. Advanced multimodal neuroimaging techniques have been employed to identify structural and functional brain changes in post-COVID patients.

There are a wide range of studies that discuss the alterations in the cerebral structure and cerebral blood flow from the post-acute phase until about 1 year from SARS-CoV-2 infection (Lu et al., 2020; Thapaliya et al., 2023; Hafiz et al., 2022; Tu et al., 2021; Douaud et al., 2022; Bendella et al., 2023; Planchuelo-Gómez et al., 2023; Cecchetti et al., 2022; Paolini et al., 2023; Heine et al., 2023; Du et al., 2022; Latini et al., 2022; Ajčević et al., 2023; Churchill et al., 2023; Qin et al., 2021; Díez-Cirarda et al., 2023), with a primary focus on hospitalized patients. However, there are very few studies investigating non-hospitalized patients with persisting symptoms more than 1 year after infection.

There is a significant heterogeneity in the reported results concerning both the specific regions affected and the type**s** of gray (GM) and white matter (WM) modifications. Studies show evidence of increased (Lu et al., 2020; Thapaliya et al., 2023; Hafiz et al., 2022; Tu et al., 2021), decreased (Douaud et al., 2022; Bendella et al., 2023; Planchuelo-Gómez et al., 2023), and unchanged (Cecchetti et al., 2022) GM volume. Similarly, changes in WM volume in different regions (Lu et al., 2020; Paolini et al., 2023; Heine et al., 2023), WM hyperintensities (Du et al., 2022; Latini et al., 2022), and decreases in perfusion in different regions (Ajčević et al., 2023) have been reported.

There is a general consensus in finding a more pronounced modification in the cortical thickness and the WM microstructure in post-COVID patients with a severe disease than in those with a mild disease (Bendella et al., 2023; Qin et al., 2021).

Many different types of alterations in functional connectivity, such as altered connectivity in the right frontal pole and in the middle-temporal gyrus (Paolini et al., 2023), have been reported in functional magnetic resonance imaging (fMRI) studies (Du et al., 2022; Churchill et al., 2023).

Only a few studies have assessed the association between brain alterations detected using magnetic resonance imaging (MRI) and cognitive impairment measured through a neuropsychological assessment (Douaud et al., 2022; Díez-Cirarda et al., 2023; Andriuta et al., 2022).

In this study, we present a multimodal neuroimaging analysis of GM, WM, brain connectivity, and perfusion-based parameters in 36 post-COVID patients and 36 individually matched controls who had mild SARS-CoV-2 infection from March 2020 to February 2022. The primary aim of this study was to investigate the structural and functional brain alterations and the secondary aim was to investigate if these alterations are associated with post-COVID symptoms, mental fatigue, and neuropsychological assessment.

## Materials and methods

### Participants

We recruited 36 post-COVID patients and 36 controls individually matched for age (±5 years) and date of COVID infection (±3 months) from April 2022 to February 2023. The post-COVID patients were enrolled at the Uppsala post-COVID outpatient clinic. The patients included in the study had COVID infection from the beginning of the pandemic through February 2022. The inclusion criteria were age between 18 and 65 years, a previous acute COVID infection that did not require hospitalization, and new onset persisting symptoms for at least 3 months after COVID infection according to the WHO definition (Soriano et al., 2022). The additional inclusion criteria were mental fatigue as one of the three most disabling symptoms, along with physical examination, radiological analysis, and blood tests ruling out other causes of the symptoms. The exclusion criteria were malignancies, autoimmune diseases, chronic diseases, and neurological, psychiatric, lung, and cardiovascular diseases prior to COVID infection.

The patients were not using antidepressants or other drugs that affect the central nervous system (CNS) at the time of recruitment such as anticonvulsants, antiemetics, CNS stimulants, muscle relaxants, narcotics, anxiolytics, or sedatives. In addition, 11 patients had COVID infection during the first wave in Sweden (February 2020–August 2020), 12 patients had COVID infection in the second wave (September 2020–February 2021), six patients in the third wave (March 2021 to July 2021), and seven in the fourth wave (August 2021–April 2022). The fourth wave was primarily driven by the omicron variant.

Every patient was individually matched to a control who had contracted COVID infection during the same period (±3 months) but experienced no post-COVID symptoms. Patients and controls has similar education levels and socioeconomic statuses, with the majority having a high level of education. The matched controls were recruited at the Östhammars primary healthcare center among patients who sought care for simple disabilities not related to COVID infection.

All the participants had a polymerase chain reaction (PCR)-verified COVID infection except for five patients and three controls who had the infection at the beginning of the pandemic when PCR capacity in Sweden was not sufficient to test all the suspected cases.

The time interval between the COVID infection and recording the brain MRI was 20.7 ± 7.6 months for patients and 23.8 ± 7.5 months for controls. The participants who were infected during the fourth wave had a shorter time interval between the infection and brain imaging (8 ± 1.7 months for patients, 11 ± 2.4 months for controls) than those who acquired the infection in the first, second, and third waves (24 ± 4.9 months for patients and 27 ± 4.6 months for controls).

The study was approved by the Swedish Ethical Review Authority (2022-01626-01) and was conducted in accordance with the Declaration of Helsinki. All participants provided their written informed consent.

### Clinical and neuropsychological assessment

The study protocol comprised assessments of physical fatigue [Fatigue Severity Scale (FSS)] (Krupp et al., 1989), mental fatigue [Mental Fatigue Scale (MFS)] (Johansson et al., 2010), depression [Montgomery Asberg Depression Rating Scale (MADRS)] (Montgomery and Asberg, 1979), and anxiety [Hospital Anxiety and Depression Scale (HAD)] (Zigmond and Snaith, 1983).

The following cut-offs adjusted for the Swedish populations were used: FSS: score range 0–63, with a cut-off of ≥36. MFS: score range 0–42, with a cut-off of ≥10. MADRS: score range 0–54, categorized as 0–12 for no depression, 13–19 for mild depression, 20–34 for moderate depression, and >35 for severe depression. HAD depression: score 0–21, cut-off ≥7. HAD anxiety: score range 0–21, with a cut-off of ≥7.

Post-COVID symptom severity was assessed using a Symptom Severity Scale (SSS) based on 17 symptoms on a 10-point scale (where 0 indicates no symptom and 10 indicates the maximum severity of the symptom). The score range is from 0 to 170.

The symptoms included in the SSS are shown in Table 1.

**Table 1:** Clinical characteristics and rating scales.

**Variable**	**Post-COVID**	**Controls**	***P*-value**
Age	44.2 ± 10.2	44.6 ± 10.5	
Gender: Women	24 (66.7%)	24 (66.7%)	
**Infection period**			
Wave 1	11 (30.6%)	13 (36.1%)	
Wave 2	12 (33.3%)	9 (25.0%)	
Wave 3	6 (16.7%)	7 (19.4%)	
Wave 4	7 (19.4%)	7 (19.4%)	
Post-COVID Symptom Severity Score	62.8 ± 24.9	8.6 ± 12.8	< 0.001
Cognitive fatigue	7.7 ± 1.9	1.1 ± 1.8	< 0.001
Physical fatigue	6.8 ± 2.2	0.9 ± 1.5	< 0.001
Dyspnea	5.1 ± 3.2	0.4 ± 1.2	< 0.001
Myalgia	4.7 ± 3.4	0.9 ± 1.4	< 0.001
Headache	4.6 ± 3.5	1.0 ± 1.5	< 0.001
Insomnia	3.9 ± 3.3	0.9 ± 1.3	< 0.001
Palpitations	3.6 ± 3.1	0.3 ± 0.8	< 0.001
Dizziness	3.2 ± 2.7	0.4 ± 1.3	< 0.001
Anxiety	3.4 ± 2.8	0.4 ± 0.9	< 0.001
Paresthesia/crawling hangindent8pt sensation	2.9 ± 3.1	0.4 ± 1.2	< 0.001
Depression	2.9 ± 2.8	0.6 ± 1.4	< 0.001
Heaviness in the hangindent8pt chest	2.8 ± 2.8	0.4 ± 1.1	< 0.001
Diarrhea/abdominal hangindent8pt pain	2.4 ± 2.8	0.4 ± 1.4	< 0.001
Tinnitus	2.3 ± 3.0	0.2 ± 0.6	< 0.001
Hyposmia/hypogeusia	2.2 ± 3.5	0.1 ± 0.5	< 0.001
Fainting	2.1 ± 2.4	0.1 ± 0.5	< 0.001
Fever	2.0 ± 3.0	0.2 ± 1.0	< 0.001
RBANS Total Index score	83.4 ± 25.1	105.2 ± 11.3	< 0.001
Immediate memory	85.0 ± 20.1	96.8 ± 15.3	0.027
Visuospatial/ hangindent8pt constructional	98.3 ± 13.8	102.4 ± 10.3	0.152
Language	91.0 ± 18.9	106.3 ± 12.7	< 0.001
Attention	81.2 ± 24.9	102.9 ± 15.1	< 0.001
Delayed memory	87.8 ± 24.9	106.8 ± 14.2	0.002
MADRS	15.7 ± 8.0	4.6 ± 4.7	< 0.001
HAD anxiety	6.5 ± 4.1	3.2 ± 2.8	< 0.001
HAD depression	7.4 ± 4.5	1.2 ± 1.5	< 0.001
FSS	55.4 ± 9.8	18.8 ± 10.6	< 0.001
MFS	22.8 ± 6.0	2.1 ± 2.6	< 0.001

Data show significant differences between patients and controls in all parameters examined except for the visuospatial/construction domain of the RBANS test. Post-COVID Symptom Severity Score (0–170) includes the 17 following symptoms on a 10-point scale (0 = no symptom, 10 = max severity). RBANS Total Scale Index and five domain-specific index scores. MADRS (depression scale 0–54), HAD anxiety (0–21), HAD depression (0–21), FSS (fatigue severity score 0–63), MFS (mental fatigue score 0–42). Data are presented as mean ± SD and compared using the Mann–Whitney U-test.

Neurocognitive status was assessed using the Repeatable Battery for the Assessment of Neuropsychological Status (RBANS) (Randolph et al., 1998; Birberg Thornberg et al., 2022). RBANS generates index scores for five neurocognitive domains, including immediate memory, visuospatial/constructional, language, attention, and delayed memory, as well as a Total Scale Index score.

All patients except two, who were not possible to reach, were followed up with a new assessment of their post-COVID Symptom Severity Score 1 year after the first test.

### Statistical analysis of clinical tests

Continuous data are represented as mean and standard deviation. Categorical data are represented as frequency and percentage.

Clinical symptoms and assessments between patients and controls were compared using the Mann–Whitney *U*-test. The correlation between MFS and RBANS was assessed using the Spearman's correlation coefficient and *p*-values were adjusted using the Bonferroni correction. The symptom scores from the first assessment and 1-year follow-up were compared using the Wilcoxon signed-rank test. Clinical symptoms and assessments between the first three waves and the fourth wave were compared using the Mann–Whitney *U*-test, and *p*-values were adjusted using the Bonferroni correction. *p*-values < 0.05 were considered statistically significant. Statistical analyses were performed using R (version 4.2.2).

### Imaging data acquisition

#### Magnetic resonance imaging

MRI scans was performed at the Uppsala University Hospital. All examinations were performed using a 3T system (Achieva d-Stream, Philips Healthcare, Amsterdam, The Netherlands). All subjects were scanned in a supine position using a 32-channel head coil. The images obtained through three-dimensional (3D) T1-weighted (3D-T1w), axial two-dimensional (2D) T2-weighted (2D-T2w), 3D fluid-attenuated inversion recovery (FLAIR), 2D axial diffusion-weighted imaging (DWI), and susceptibility-weighted imaging (SWI) were acquired for morphological evaluation and volumetric analysis. Perfusion-weighted imaging included a 3D pseudo-continuous arterial spin labeling (pCASL) sequence with gradient spin-echo readout and dynamic susceptibility contrast (DSC) acquisition. Additionally, resting-state fMRI (rs-fMRI) and diffusion tensor imaging (DTI) were acquired as part of the imaging protocol. A full description of the imaging protocol is provided in Supplementary Table 1.

### Morphological evaluation

Two certified neuroradiologists (JW and DF) performed independent evaluations regarding WM changes, cortical infarcts, lacunar infarcts, microbleeds, and global atrophy. WM changes were assessed using the FLAIR images and categorized according to the Fazekas scale (0–III) (Fazekas et al., 1987). The number of cortical infarcts, lacunar infarcts, and microbleeds were noted; the infarcts were assessed using mainly FLAIR and 2D-T2-weighted sequences, and microbleeds were assessed in the SWI images. When microbleeds were present, they were assessed using a slightly modified Medication Adherence Rating Scale (MARS) (Gregoire et al., 2009) (using the SWIp sequence instead of GRE T2^*^ and not recording laterality).

The width of the cerebral sulci was assessed using the global cerebral atrophy (GCA) scale (Pasquier et al., 1996).

The differences in routine morphological measures were compared between patients and controls using chi-squared or Fisher tests as appropriate, utilizing SPSS (version 28.0.1.0, IBM SPSS, Armonk, NY). A *p*-value of < 0.05 was regarded as statistically significant.

### Volumetry

The 3D-T1w images of all the subjects were processed with FreeSurfer software (http://surfer.nmr.mgh.harvard.edu/, version 7.4.0) using the *recon-all* pipeline. GM volumes and cortical thickness were extracted for the following cortical and subcortical regions: the frontal, parietal, occipital, and temporal lobes (see Supplementary Table 2 for components), cerebellum, hippocampus, putamen, pallidum, and thalamus. In addition, total cerebral gray matter, total cerebral white matter, and total cerebellar white matter were extracted.

### Perfusion assessment

The pCASL-based cerebral blood flow (CBF) maps (CBF_pCASL_) were automatically calculated by the scanner according to the model recommended by Alsop et al. (2015). Based on the DSC acquisition, parametric maps of CBF, cerebral blood volume (CBV), mean transit time (MTT), time to peak (TTP), leakage, coefficient of variation (CoV), capillary transit time heterogeneity (CTH), cerebral metabolic rate of oxygen (CMRO_2_), and oxygen extraction fraction (OEF) were calculated using Cercare Medical Neurosuite (Cercare Medical, Aarhus, Denmark). Parametric maps were registered to the corresponding 3D-T1w image. The perfusion-based measures as listed above were extracted from cortical and subcortical regions derived from FreeSurfer as described above.

### Statistical analysis of volumetry and perfusion assessment

Hypothesis testing was focused on the derived regional parameters, that is, perfusion-based metrics, cortical volumes, and thicknesses, comparing the measures between controls and patients. First, the Shapiro–Wilk test was performed to test for normality. To test whether any differences were present between controls and patients, we performed a Kruskal–Wallis test for non-normal distributed data with Dunn's correction for multiple comparisons. The *post-hoc* analysis was performed for all the cortical regions for each parameter.

### Voxel-based morphometry analysis

The voxel-based morphometry (VBM) analysis of the gray matter was performed in FSL (the FMRIB Software Library) toolbox (https://fsl.fmrib.ox.ac.uk) version 6.0.6.1 using the standard processing pipeline FSL_VBM as described in detail (Jenkinson et al., 2012).

(i) A group comparison was performed for patients vs. controls using age and gender as non-explanatory co-regressors.
(ii) The analysis of patients vs. controls was conducted using age and gender as non-explanatory co-regressors, adding “time between COVID infection and MRI” as an additional non-explanatory co-regressor.
(iii) A group comparison was performed for patients vs. controls using only the first, second, and third waves again using age and gender as non-explanatory co-regressors.
(iv) A correlation analysis was performed using the three most relevant test scores, notably RBANS total scale index score, MFS, and SSS, again using age and gender as non-explanatory co-regressors.

All variables were transformed into the range of −1 to +1 with a mean of 0. A statistical threshold was defined as *p* < 0.05 corrected using threshold-free cluster enhancement (TFCE) (Smith and Nichols, 2009).

### Track-based spatial statistics

The track-based spatial statistics (TBSS) analysis of the white matter was performed in FSL version 6.0.6.1 using the standard processing pipeline FSL_TBSS as described in detail (Jenkinson et al., 2012). The studied parameters related to the white matter integrity included fractional anisotropy (FA), mean diffusivity, and axial and radial diffusivity. Four equivalent analyses were performed similar to the VBM analysis described above. Again, a statistical threshold of TFCE-corrected *p* < 0.05 was applied.

### Resting fMRI analysis

Resting fMRI analysis was performed in FSL version 6.0.6.1 using the standard processing pipeline MELODIC as described in detail (Jenkinson et al., 2012). First, a tensorial independent component analysis (TICA) was performed using 20 independent components. Then, a dual regression analysis was performed using the same setup as above: first, a group comparison between patients and controls was carried out and then a correlation analysis was performed with the same three clinical scores, notably RBANS Total Scale Index score, MFS, and SSS, again using age and gender as non-explanatory co-regressors and a statistical threshold of TFCE-corrected *p* < 0.05.

## Results

### Clinical characteristics

The detailed clinical information regarding the date of COVID infection and responses to rating scales and neurocognitive tests are reported in Supplementary Table 3. The participants were aged between 24 and 64 years. The mean age of COVID patients was 44.2 ± 10.2 years and that of controls was 44.6 ± 10.5 years. The majority (66.6%) of the patients in both groups were women (Table 1).

The post-COVID Symptom Severity Score (values ranging between 0 and 170) was 62.8 ± 24.9 for patients and 8.6 ± 12.8 for controls (*p* < 0.001).

The patient-reported complaints with the highest mean value were cognitive fatigue (7.7 ± 1.9), physical fatigue (6.8 ± 2.2), and dyspnea (5.1 ± 3.2; Table 1).

The patients fulfilled the criteria for mild depression (MADRS 15.7 ± 8.0, HAD depression 7.4 ± 4.5) and were just below the cut-off score for anxiety (HAD anxiety 6.5 ± 4.1). The levels of depressive symptoms and anxiety were anyway higher (*p* < 0.001) compared to healthy controls (Table 1).

They reported higher levels of both physical fatigue (FSS 55.4 ± 9.8) and mental fatigue (MFS 22.8 ± 6.0) compared to healthy controls (*p* < 0.001).

Neurocognitive testing showed a lower RBANS Total Scale Index score in patients (*p* < 0.001). Differences were found between patients and controls in attention and language (*p* < 0.001), immediate memory (*p* = 0.027), and delayed memory domains (*p* < 0.002), whereas no difference was observed in visuospatial/construction domain (*p* < 0.152).

Subjective mental fatigue assessed with the MFS test correlated with objective cognitive impairment measured with the RBANS score (*r* = −0.46, *p* < 0.05, Table 2). Particularly, patients with higher levels of subjective mental fatigue had lower performance in the attention domain (*r* = −0.56, *p* = 0.002; Table 2). The other four RBANS domains did not correlate with the subjective mental fatigue.

**Table 2:** Correlation between the mental fatigue score (MFS) and the neurocognitive test (RBANS).

**Scale**	**Corr. coef**.	***P*-value**
RBANS Total Index score	−0.46	0.027
Immediate memory	−0.37	0.15
Visuospatial/constructional	−0.36	0.18
Language	−0.16	>0.99
Attention	−0.56	0.002
Delayed memory	−0.36	0.19

Spearman's correlation coefficient. The *p*-values were adjusted using the Bonferroni correction for multiple comparisons across six tests.

MFS score is strongly negatively correlated to the attention domain of the RBANS test.

When we compared patients who contracted the omicron variant (fourth wave) with patients with earlier COVID variants, there was no significant difference between the groups in symptoms, neurocognitive ability, and responses to rating scales (Supplementary Table 4).

One year after the first assessment, 34 patients were re-evaluated with the post-COVID Symptoms Assessment Scale (SAS). Post-COVID Symptom Severity Score decreased from 62.7 ± 25.6 at the first medical visit to 47.3 ± 26.7 after 1 year (*p* < 0.001). Self-reported cognitive fatigue was also found to be improved (from 7.7 ± 1.9 at the first visit to 6.2 ± 2.5 after 1 year, *p* < 0.001). However, when asked to estimate their health in relation to post-COVID symptoms, only two patients reported feeling completely recovered (Supplementary Table 3).

### Routine morphological MRI evaluation

Both reviewers observed that there were no significant differences in the number of cortical infarcts, lacunar infarcts, white matter hyperintensities, global cerebral atrophy, or microbleeds between patients and controls (Table 3).

**Table 3:** Macrostructural analysis.

**Fazekas**	**Reviewer 1**			**Reviewer 2**		
	**Grade 0**	**Grade 1**	**Grade 2**	**Grade 0**	**Grade 1**	**Grade 2**
Controls	8	25	3	15	19	2
Patients	8	25	3	18	16	2
	*p* = 1.00			*p* = 0.77		
**GCA**	**Grade 0**	**Grade 1**	**Grade 2**	**Grade 0**	**Grade 1**	**Grade 2**
Controls	2	32	2	29	7	0
Patients	2	32	2	35	1	0
	*p* = 1.00			*p* = 0.972		
**Cortical infarcts**	**0**	**1 or more**	**0**	**1 or more**
Controls	35	1	36	0
Patients	34	2	36	0
	*p* = 0.5		NA	
**Lacunar infarcts**	**0**	**1 or more**	**0**	**1 or more**
Controls	36	0	35	1
Patients	32	4	35	1
	*p* = 0.057		*p* = 0.75	
**Microbleeds**	**0**	**1 or more**	**0**	**1 or more**
Controls	32	4	35	1
Patients	32	4	34	2
	*p* = 0.65		*p* = 0.5	

Comparisons of white matter changes (Fazeka), global cerebral atrophy (GCA), cortical infarcts, lacunar infarcts, and microbleeds between controls and patients for both reviewers. No significant differences were observed.

### MR volumetry

**Figure 1:**
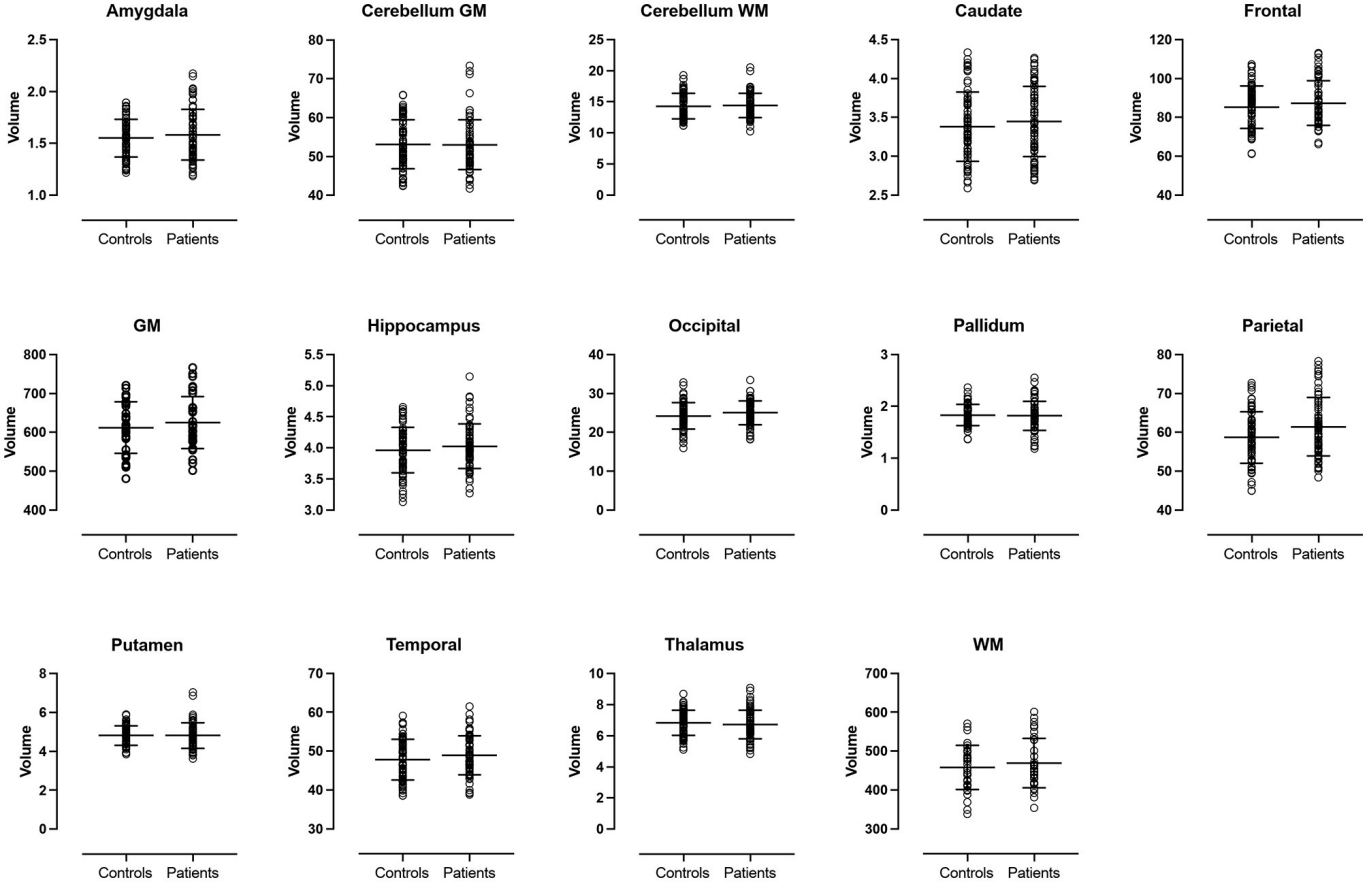
Volume analysis. Volumes of the cerebral lobes, cerebral central gray matter structures and cerebellum. No significant differences were observed between patients and controls. GM, total cerebral gray matter. WM, total cerebral white matter.

**Figure 2:**
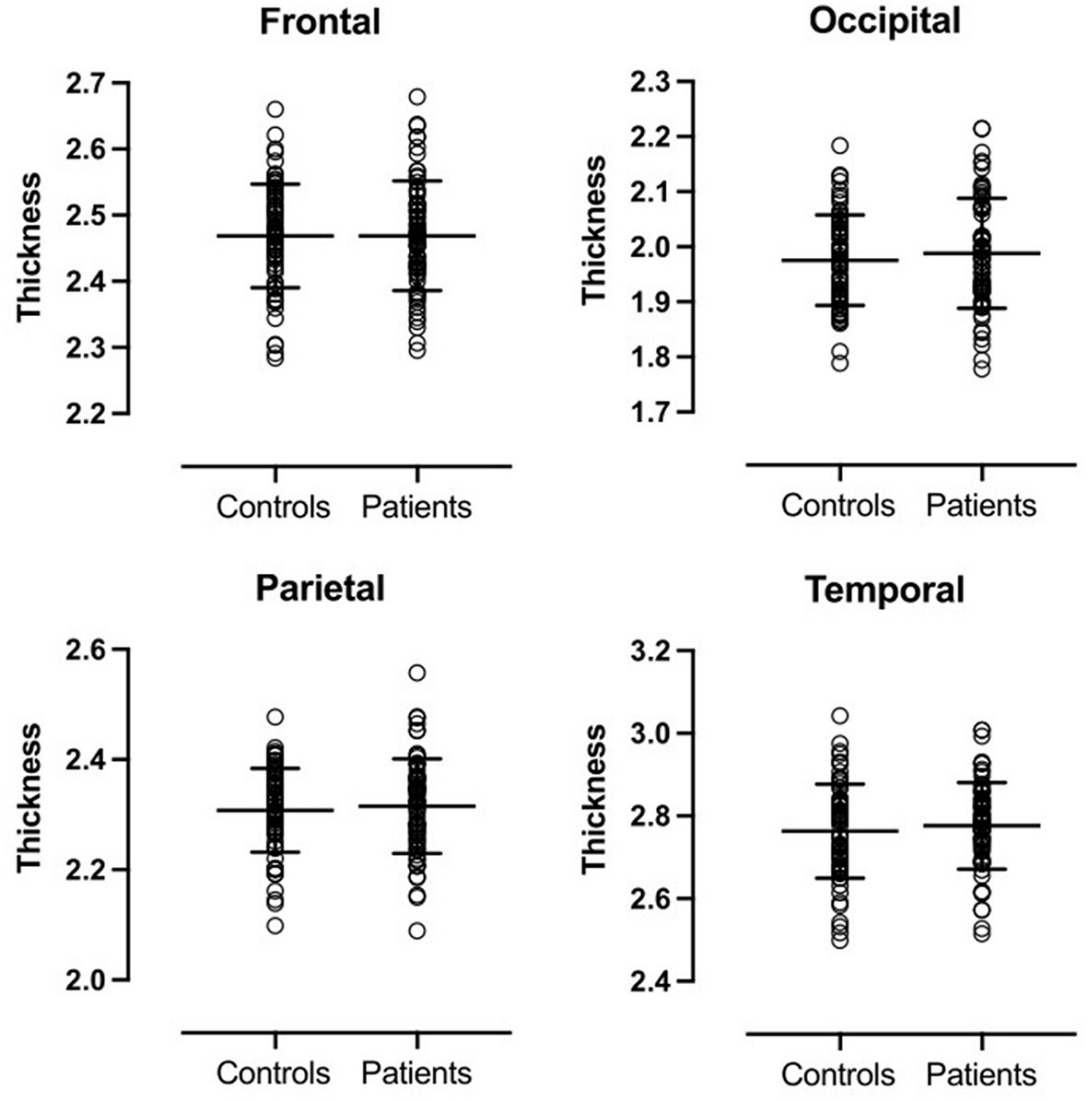
Cortical thickness of cerebral lobes. No significant differences were observed between patients and controls.

There were no significant differences in volume or cortical thickness in any of the anatomical regions between patients and controls (Figures 1, 2).

### MR perfusion

There were no significant differences in CBF_pCASL_, CBF, CBV, MTT, TTP, leakage, CV, CTH, CMRO_2_, or OEF in any of the anatomical regions between patients and controls (Supplementary Figures 1–10).

### Voxel-based morphometry analysis

**Figure 3:**
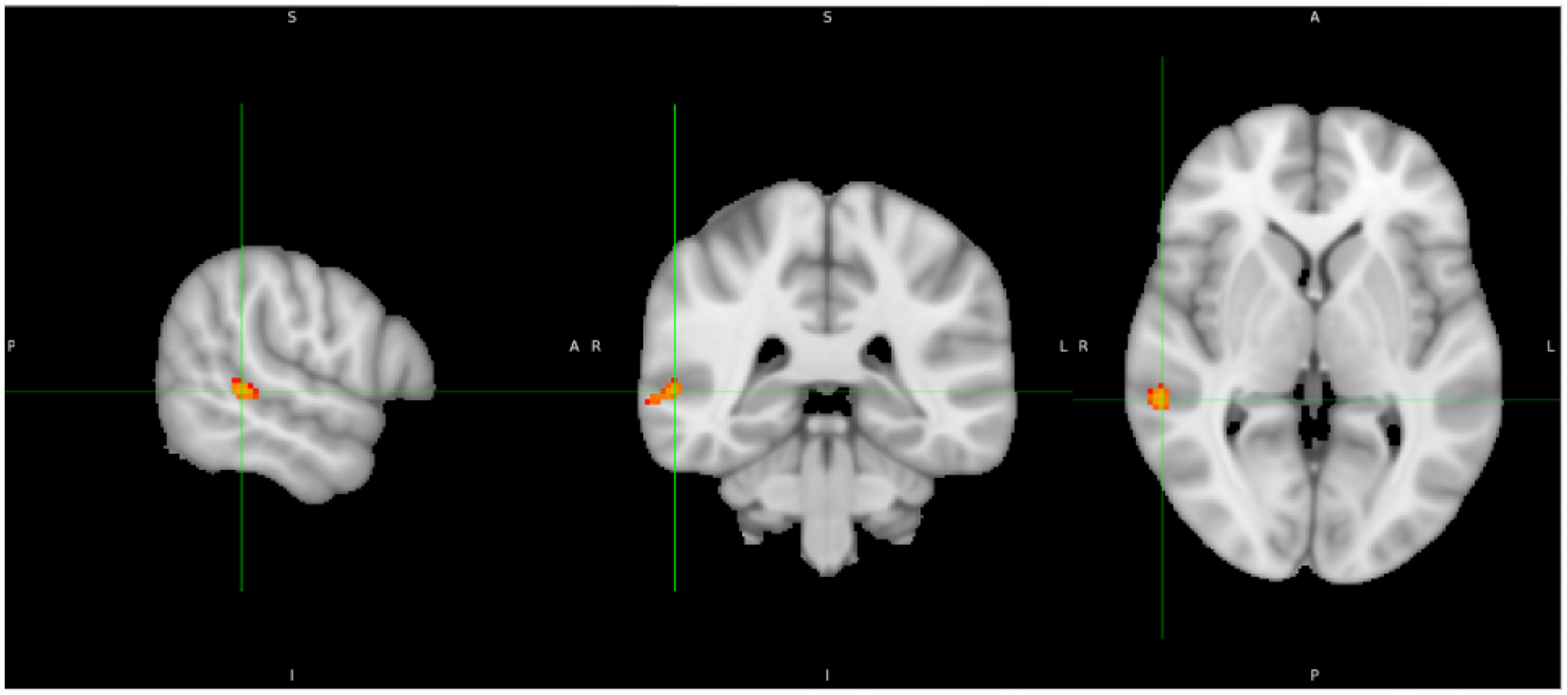
Correlation analysis shows a positive correlation between gray matter volume and RBANS Total Index score in the posterior part of the right superior-/middle-temporal gyrus (yellow).

(i) There was no significant group difference between patients and controls in gray matter VBM.
(ii) Adding “time between COVID infection and MRI” as an additional co-regressor did not change those results.
(iii) The comparison between patients and controls exclusively in the first, second, and third waves (early variants) resulted still in no significant differences.
(iv) In the whole group, we observed a significant positive correlation with RBANS total index score (*p* < 0.05 TFCE-corrected) for gray matter volume in the right superior/middle-temporal gyrus (Figure 3).

### Track-based spatial statistics

**Figure 4:**
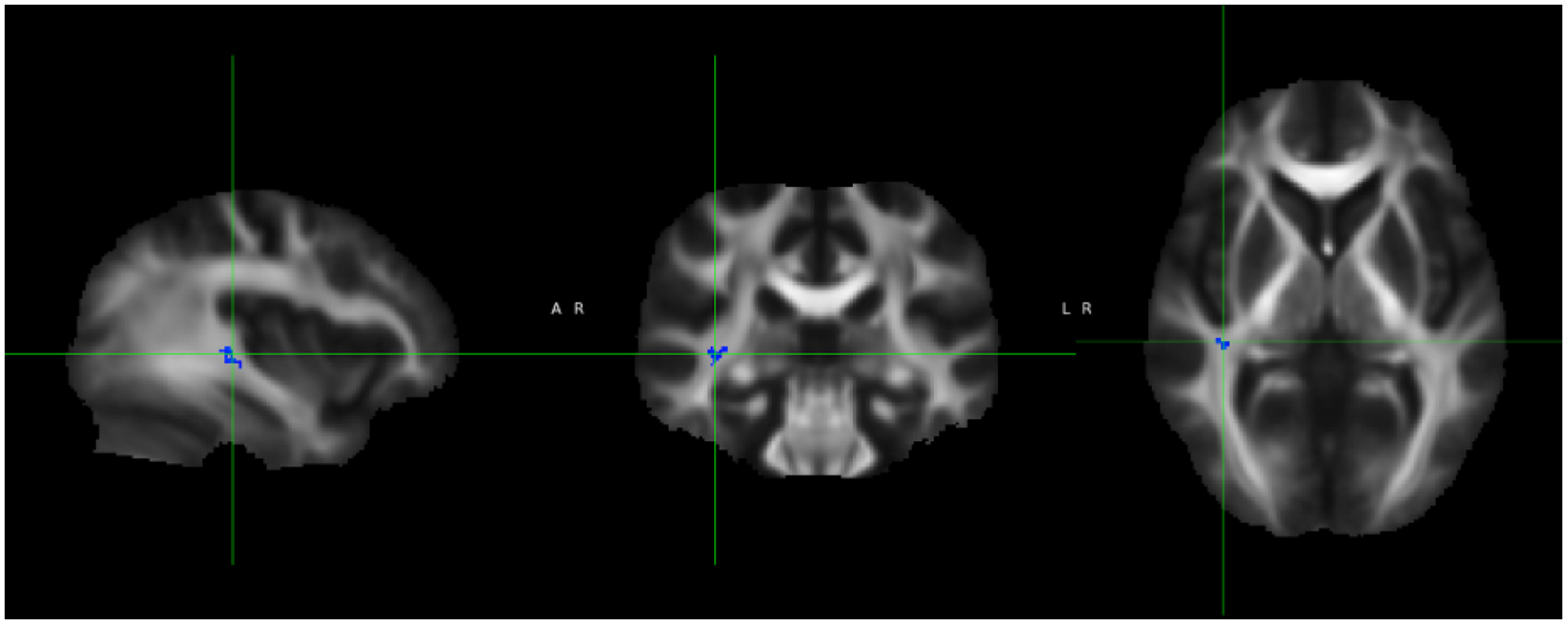
Track-based spatial statistics shows a significant negative correlation between Symptom Severity Score (SSS) and FA in the right superior-/middle-temporal gyrus (blue).

(i) There was no significant group difference between patients and controls in the white matter TBSS analysis.
(ii) Adding “time between COVID infection and MRI” as an additional co-regressor did not change the results.
(iii) Again, analysis of patients and controls in only the first, second, and third waves (early variants) resulted in no significant differences.
(iv) In the whole group, we observed a significant negative correlation between FA in the right superior-/middle-temporal gyrus and the SSS (TFCE-corrected *p* < 0.05; Figure 4).

### Resting fMRI analysis

**Figure 5:**
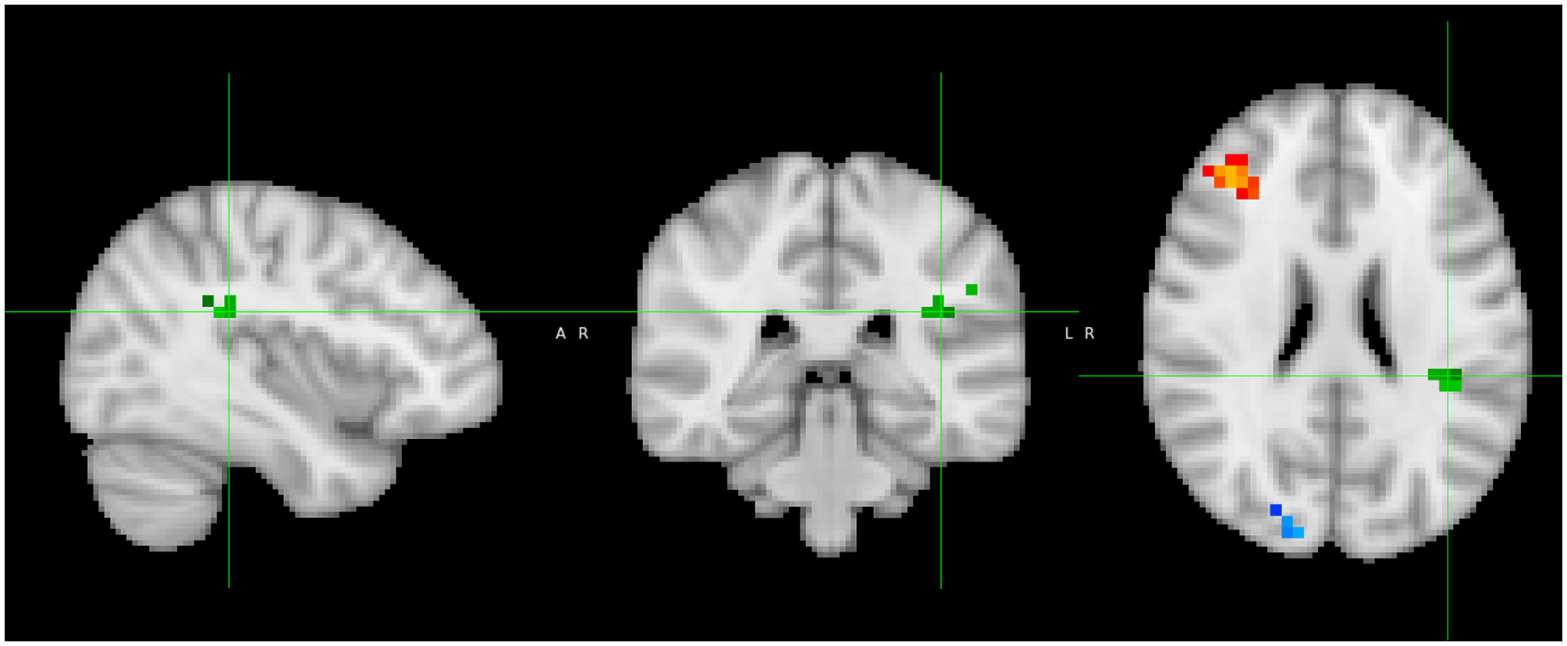
Dual regression resting state fMRI analysis shows stronger connectivity in the right middle frontal gyrus (orange) in patients compared to controls and weaker connectivity in the right inferior parietal lobule (blue) and left fronto-parietal junction (green).

Based on dual regression, patients had a significantly stronger connectivity in the right middle frontal gyrus (MFG) compared to controls, while they exhibited a significantly weaker connectivity in the right inferior parietal lobule and the left fronto-parietal junction compared to controls (see Figure 5).

**Figure 6:**
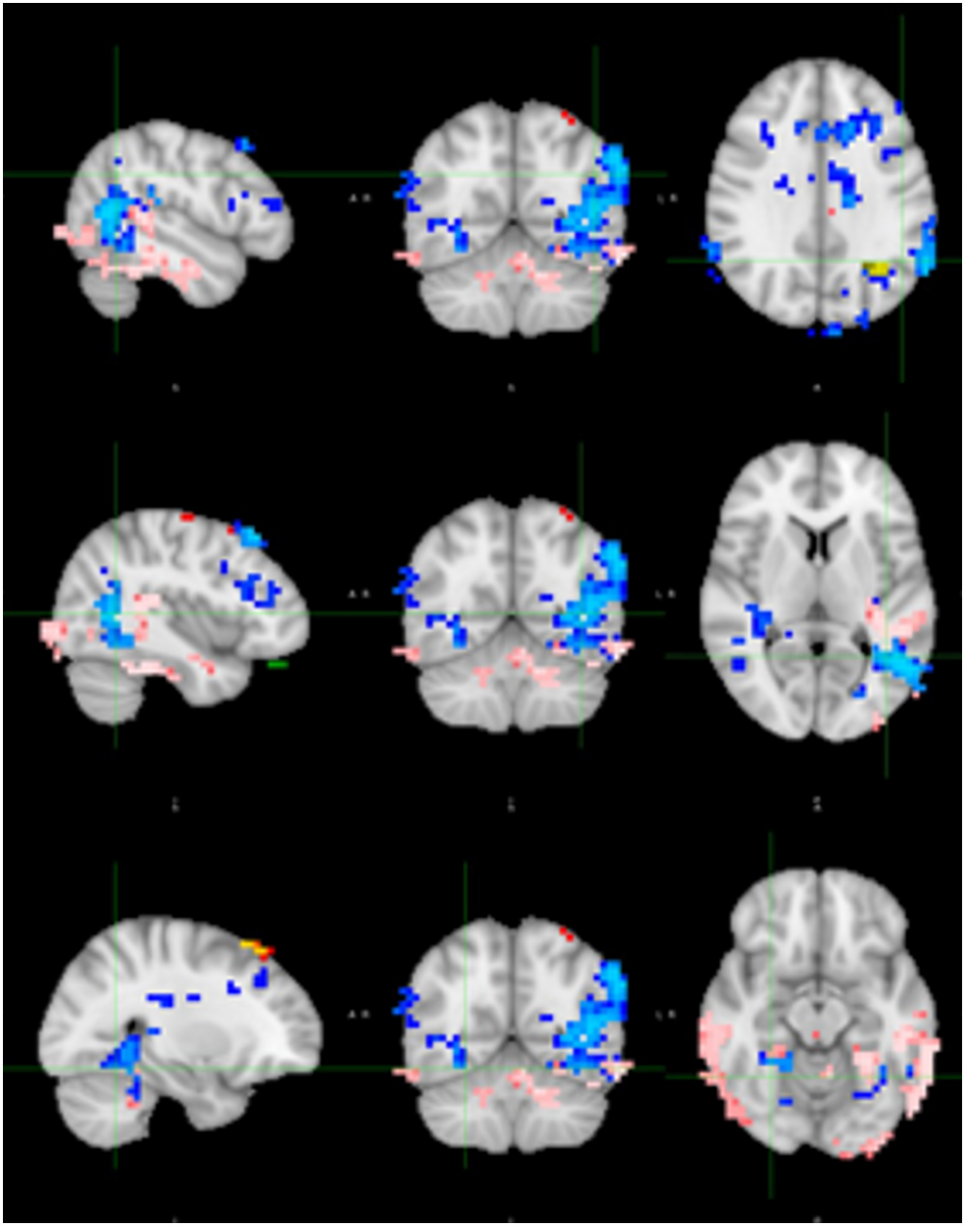
Correlation between SSS and connectivity. Positive correlation in the right posterior temporoparietal junction (TPJ; blue) and bilateral temporo-occipital junction (pink), and weaker correlations in the left frontobasal (green) and left superior parietal (red) areas. There was a minor negative correlation in the left parietal region (yellow).

Additionally, we observed a significantly positive correlation in the whole group using the SSS, notably in the right posterior temporoparietal junction (TPJ) and bilateral temporo-occipital junction, and weaker correlations in the left frontobasal and left superior parietal areas were observed. There was a minor negative correlation in the left parietal region (Figure 6).

## Discussion

The aim of the present study was to use a multimodal imaging approach to evaluate brain alterations in post-COVID patients after a mild COVID infection and their correlation with post-COVID symptoms (SSS), mental fatigue (MFS), and neuropsychological assessments (RBANS test). We found no significant morphological, microstructural, or perfusion brain changes in non-hospitalized post-COVID patients with brain fog who were treated at an outpatient post-COVID clinic in comparison with age-matched healthy controls who had acquired COVID infection in the same period. However, some differences were observed between the two groups regarding functional brain connectivity, particularly in the right middle frontal gyrus, which may suggest a disturbed regulation mechanism between ventral and dorsal attention networks.

### Macro- and microstructure and perfusion

We found no differences in macrostructural abnormalities, such as infarcts and atrophy, microbleeds, number, and localization of WM hyperintensities and general brain volume. The absence of differences in focal lesions is in agreement with a recent review of routine MR imaging studies, which found minimal abnormalities in post-COVID subjects and no conclusive evidence of a correlation between MRI findings and symptoms (Vasilev et al., 2023). As for brain volumes, previous studies have shown divergent results (Lu et al., 2020; Bendella et al., 2023).

Regional perfusion measures were not different in our study between post-COVID subjects and controls. Apart from CBF measurements, we also used novel perfusion-based measures such as CTH, OEF, and CMRO_2_ (Mouridsen et al., 2014) for an assessment of microvascular derangements. We found no evidence of alterations in post-COVID patients.

Regional GM density and regional WM fractional anisotropy were also similar between the patient groups. The absence of microstructural white matter changes in the whole brain is mostly in line with the findings of Heine et al. (2023) who compared 47 subjects with post-COVID symptoms with subjects without COVID infection although they found aberrant FA of the thalamus. Paolini et al. (2023) also failed to identify any changes in fractional anisotropy but, on the other hand, they showed increases in diffusivity measures in several areas in patients previously hospitalized because of COVID infection, which can be interpreted as an increase in water content. The differences in results may be explained by both differences in infection severity (hospitalized vs. non-hospitalized) and choice of the comparison group (without a medical history of COVID infection vs. with a medical history of COVID infection but without remaining symptoms).

### Connectivity

The only positive findings were differences in resting-state functional connectivity, where post-COVID patients showed higher connectivity in the right middle frontal gyrus. Altered connectivity in a similar frontal region in post-COVID patients was also detected by Paolini et al. (2023). The right middle frontal gyrus has been proposed to be the node that links the ventral and dorsal attention networks by acting as a “circuit-breaker” between exogenous and endogenous attention control (Corbetta et al., 2008). Fox et al. (2006) have shown that the right middle frontal gyrus interrupts ongoing processes in the dorsal network and reorients attention to a novel task-relevant external stimulus. Impaired connectivity in the right MFG and the resulting loss of the flexible modulation between endogenous and exogenous attention may eventually be responsible for certain post-COVID symptoms such as difficulties with focus and concentration in a specific task, as well as oversensitivity to minimal external stimuli, which are perceived as distracting and increasing the mental exhaustion.

This hypothesis is supported by the fact that we additionally found a positive correlation between post-COVID symptoms and increased connectivity in the right TPJ, which is the main part of the ventral attentional system. The increased connectivity in the ventral network in participants with post-COVID symptoms may reflect their inability to filter distracting signals.

### Correlation between the neuropsychological test and subjective mental fatigue

Post-COVID patients showed cognitive impairment in all RBANS domains except visuospatial/construction. These results are in accord with another study in which a neurocognitive test was performed using Montreal Cognitive Assessment (MoCA) and where no differences in orientation and visuoconstructive functions were observed (Birberg Thornberg et al., 2022).

The patients who reported a higher value of subjective mental fatigue at the MFS also had a more pronounced objective impaired cognitive ability at the RBANS test, especially in the Attention domain. The fact that impaired attention ability is the neurocognitive domain that correlates with the subjective feeling of mental exhaustion in our patients reinforces the hypothesis that deficit in attention mechanisms can be the basis of the subjective mental fatigue in post-COVID patients and it is in line with our results in connectivity.

### Correlation between the neuropsychological test and MRI

We found a positive correlation between neurocognitive test and GM thickness in the right superior-/middle-temporal gyrus. Interestingly, there was also a correlation between the intensity of post-COVID symptoms (SSS) and WM anisotropy in the same area. These findings are difficult to interpret but imply that this region is involved in both cognitive processes and post-COVID symptoms. Neurocognitive impairment did not correlate with WM anisotropy, similar to a previous study in 86 post-COVID patients (Díez-Cirarda et al., 2023).

### Time since infection

Another point of interest to consider is the time of infection. Patients in our study acquired SARS-CoV-2 infection from the beginning of the pandemic in 2020 to February 2022. This period includes four waves of the pandemic in Sweden, where the fourth wave was dominated by the omicron variant. The participants who had the infection in the first three waves were not vaccinated, while those who contracted omicron (the fourth wave) were vaccinated with at least two shots. To assess whether individuals who contracted the virus before receiving the vaccination—during a period when the virus was more aggressive—could have more pronounced structural brain changes, we separately analyzed patients and controls in the first three waves but found no structural differences in brain changes.

There was also no difference in post-COVID symptoms, neurocognitive ability, depression, anxiety, and physical and mental fatigue between the patients who had contracted the infection in the first three waves and those who were infected by the omicron variant. However, the small number of patients in the omicron group may not be sufficient to detect a difference between the two groups (omicron and “pre-omicron”). Therefore, these data must be interpreted with caution.

We did not find any correlation between brain changes and the time passed between infection and MRI.

Except for the participants who were infected with the omicron variant (a minority), our patients had experienced post-COVID symptoms for a long time before undergoing MRI (24 ± 5 months), which is longer compared to most other studies.

After 2 years of illness, one patient in our study was diagnosed with myalgic encephalomyelitis/chronic fatigue syndrome (ME/CFS), and others were under investigation since most of the symptoms reported by individuals with ME/CFS and post-COVID are similar. According to evidence from a prospective study, it appears that the subset of post-COVID patients who also fulfill the diagnostic criteria for ME/CFS presents more persistent, high-severity symptoms at a 20-month follow-up, while patients affected by COVID show an overall health improvement in the post-COVID phase (Legler et al., 2023).

In our study, when we reassessed the patients using the post-COVID Symptoms Assessment Scale (SAS) after 1 year, all of them had a lower intensity of symptoms, although only two felt fully recovered; there was thus a trend toward slow improvement.

Interestingly, the localization of the impaired brain connectivity seems different between patients with ME/CFS and post-COVID patients. In ME/CFS, connectivity seems impaired within the brainstem and from the brainstem to key subcortical structures. Moreover, hippocampal connections to the midbrain cuneiform nucleus and the medulla are enhanced, suggesting that the hippocampus has a compensatory role for the impaired connections (Barnden et al., 2019; Inderyas et al., 2024). In our post-COVID study, we could not find any impairment in the brainstem connectivity, suggesting that different brain areas are possibly affected.

### Strengths and limitations

A strength of the study is the stringent inclusion criteria for controls. The higher proportion of women (66.6%) in our study reflects the higher prevalence of post-COVID symptoms in women in the general population.

Another strength is the comprehensive MR protocol, including macrostructural, microstructural, perfusion, and cortical activation outcomes. The perfusion protocol included an assessment of capillary transit time heterogeneity, which is a recently introduced metric with the potential to provide significant insights into microvascular function. However, a potential weakness is that the statistical power of the present study may not have been sufficient to detect small structural differences between the study groups. In particular, the results of the subgroup analysis (omicron variant vs. “pre-omicron” variant) can be affected by the low number of patients in the fourth wave.

### Potential relevance in clinical practice

Our study presents a hypothesis (post-COVID brain fog is related to attention problem, which in turn is related to impaired connectivity in the right MFG and increased connectivity in the ventral network) that has potential high relevance in clinical practice and future research. This hypothesis needs to be confirmed in larger studies. Additionally, the negative finding concerning the absence of morphological, microstructural, or perfusion brain changes is relevant in clinical practice to avoid unnecessary brain MRI examinations in post-COVID patients, since connectivity studies are not included in the routine MRI examination.

## Conclusion

This study found no macrostructural or microstructural brain changes, nor alterations in perfusion, in patients who exhibited persistent subjective and objective impaired cognitive abilities long after a mild COVID infection. However, differences in functional connectivity were observed in a few regions, notably the right medial frontal gyrus, which is an important area for attention processes. These changes could play a role in post-COVID brain fog. However, the results of our exploratory analysis in this region require confirmation through larger studies.

## Data Availability

The raw data supporting the conclusions of this article will be made available by the authors, without undue reservation.
