# Supplementary material for: Comprehensive MRI assessment reveals subtle brain findings in non-hospitalized post-COVID patients with cognitive impairment
Source: Front Neurosci. 2024 Sep 10;18:1435218. doi: 10.3389/fnins.2024.1435218 (PMC11420131; doi:10.3389/fnins.2024.1435218)
Supplement: Supplementary file 1 [file Table_1.docx]

# Supplementary Table 1. Sequence parameters of MR imaging protocol

| **Sequence** | **Type** | **Orientation** | **Spatial resolution (mm)** | **TR (ms)/TE (ms)/flip angle (degree)** | **NSA** | **b values (s/mm^2^)** | **Diffusion sensitizing directions** |
| --- | --- | --- | --- | --- | --- | --- | --- |
| T1 weighted | 3D turbo gradient echo | sagittal | 0.87 x 0.87 x 1 | 8.9/4.1/8 | 3 | NA | NA |
| T2 weighted | 2D turbo spin echo | axial | 0.4 x 0.4 x 4 | 3000/80/90 | 1 | NA | NA |
| SWI | 3D gradient echo | axial | 0.3 x 0.3 x 4 | 31/0/17 | 1 | NA | NA |
| FLAIR | 3D IR-turbo spin echo | sagittal | 0.6 x 0.6 x 1 | 4800/334/90 | 1 | NA | NA |
| Diffusion weighted | 2D echo planar imaging | axial | 0.9 x 0.9 x 4 | 3601/75/90 | 1 | 0 and 1000 | 3 |
| ASL perfusion | 3D pCASL | axial | 3 x 3 x 6 | 4173/10.7/ 90 | 1 | NA | NA |
| DSC perfusion | 2D gradient echo | axial | 1.7 x 1.7 x 4 | 1298/29/90 | 1 | NA | NA |
| DTI | 2D | axial | 1.75 x 1.75 x 2 | 3739/86/90 | 1 | 0 and 1000 | 48 |
| rs f-MRI | 2D gradient echo | axial | 1.88 x 1.88 x 2.8 | 2000/30/90 | 1 | NA | NA |
