# Supplementary material for: Comprehensive MRI assessment reveals subtle brain findings in non-hospitalized post-COVID patients with cognitive impairment
Source: Front Neurosci. 2024 Sep 10;18:1435218. doi: 10.3389/fnins.2024.1435218 (PMC11420131; doi:10.3389/fnins.2024.1435218)
Supplement: Supplementary file 2 [file Table_2.docx]

**Supplementary Table 2:** Components of cerebral lobes used in volumetric analysis, based on the Desikan-Killiany atlas.

| **Frontal**  Caudal Anterior Cingulate  Caudal Middle Frontal  Lateral Orbitofrontal  Medial Orbitofrontal  Paracentral  Pars Opercularis  Pars Orbitalis  Pars Triangularis  Precentral  Rostral Anterior Cingulate  Rostral Middle Frontal  Superior Frontal  Frontal Pole | **Occipital**  Cuneus  Lateral Occipital  Lingual  Pericalcarine |
| --- | --- |
| **Temporal**  Entorhinal  Fusiform  Inferior Temporal  Middle Temporal  Parahippocampal  Superior Temporal  Temporal Pole  Transverse Temporal | **Parietal**  Inferior Parietal  Isthmus  Postcentral  Posterior Cingulate  Precuneus  Superior Parietal  Supramarginal |
