# Supplementary material for: Comprehensive MRI assessment reveals subtle brain findings in non-hospitalized post-COVID patients with cognitive impairment
Source: Front Neurosci. 2024 Sep 10;18:1435218. doi: 10.3389/fnins.2024.1435218 (PMC11420131; doi:10.3389/fnins.2024.1435218)
Supplement: Supplementary file 4 [file Table_4.docx]

**Supplementary Table 4**. Omicron variant (4th wave) vs other variants (1st-2nd-3th waves)

| **Scale** | **Omicron** | **Other variants** | **P-value** |
| --- | --- | --- | --- |
| RBANS | 97.0 ± 16.4 | 80.1 ± 25.9 | 0.39 |
| Immediate Memory | 100.7 ± 8.9 | 81.2 ± 20.3 | 0.19 |
| Visuospatial/Constructional | 101.1 ± 7.9 | 97.6 ± 14.9 | >.99 |
| Language | 98.4 ± 13.1 | 89.2 ± 19.8 | >.99 |
| Attention | 86.3 ± 18.7 | 80.0 ± 26.3 | >.99 |
| Delayed Memory | 103.0 ± 18.2 | 84.1 ± 25.2 | 0.34 |
| MADRS | 14.7 ± 8.6 | 15.9 ± 8.0 | >.99 |
| HAD anxiety | 7.3 ± 4.3 | 6.3 ± 4.1 | >.99 |
| HAD depression | 7.0 ± 3.5 | 7.5 ± 4.7 | >.99 |
| FSS | 52.3 ± 17.0 | 56.1 ± 7.4 | >.99 |
| MFS | 20.6 ± 8.4 | 23.3 ± 5.3 | >.99 |
| Symptom severity | 62.9 ± 23.3 | 62.8 ± 25.7 | >.99 |

The p-values obtained from the Mann-Whitney U test were adjusted using the Bonferroni correction for multiple comparisons across 12 tests.

There is no difference in symptoms and responses to rating scales between patients who contracted COVID early (wave 1-2-3) and those who contracted the omicron variant (wave 4).
