# Supplementary material for: Comprehensive MRI assessment reveals subtle brain findings in non-hospitalized post-COVID patients with cognitive impairment
Source: Front Neurosci. 2024 Sep 10;18:1435218. doi: 10.3389/fnins.2024.1435218 (PMC11420131; doi:10.3389/fnins.2024.1435218)
Supplement: Supplementary file 5 [file Data_Sheet_1.docx]

**Supplementary Figure 1-10.** Comparison of **perfusion** parameters between patients and controls. No significant differences were observed in any of the anatomical regions. Abbreviations: GM: grey matter, WM: white matter, CBF_ASL: cerebral blood flow measured with arterial spin labeling, CBF: cerebral blood flow, CBV: cerebral blood volume, MTT: mean transit time, TTP: time-to-peak, CTH: capillary transit time heterogeneity, COV: coefficient of variation, CMRO2: cerebral oxygen metabolic rate, OEF: oxygen extraction fraction.


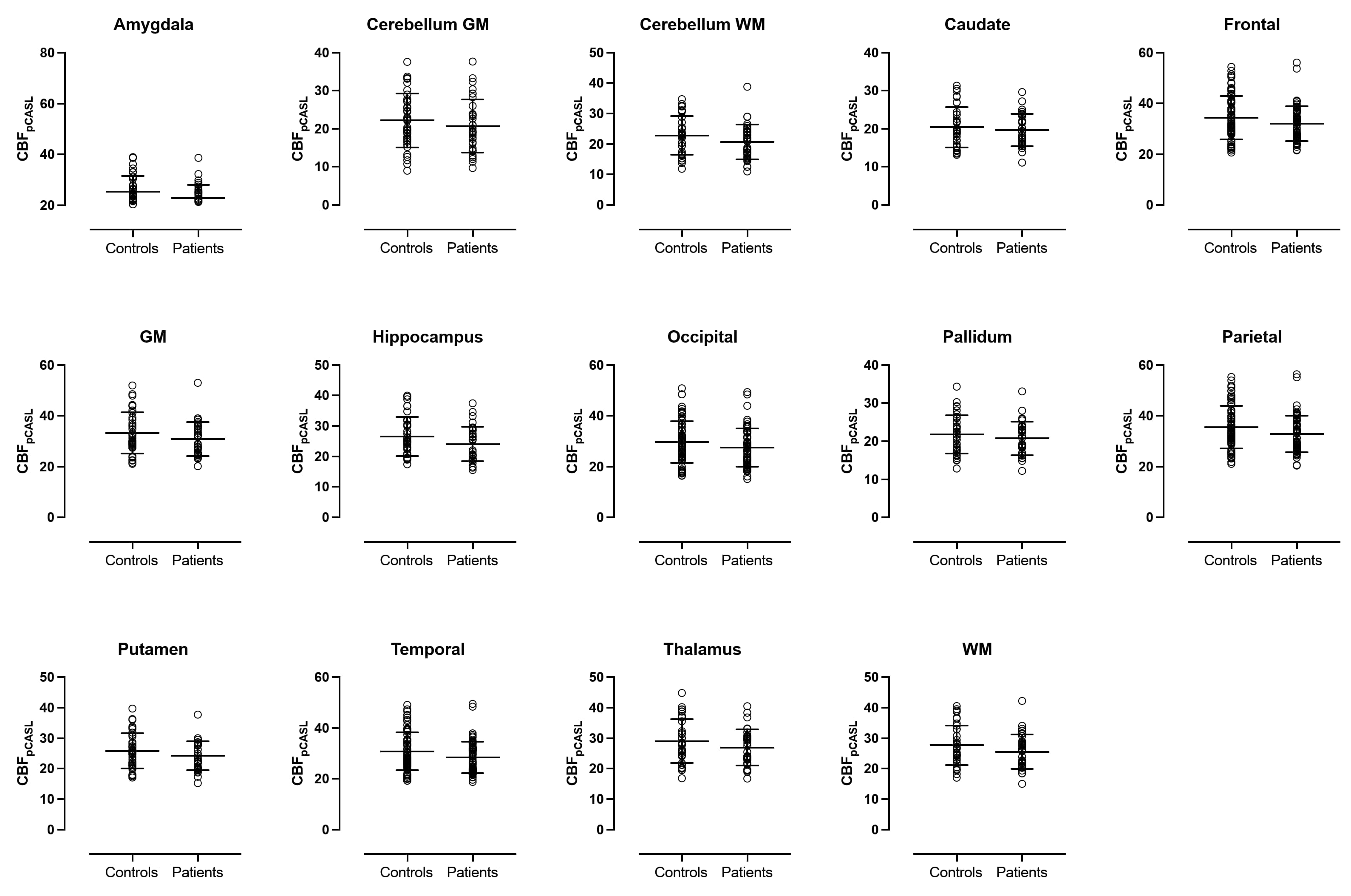


**Supplementary Figure 1:** Scatter plots for ASL-based cerebral blood flow (CBF_pCASL_) in cortical- and subcortical regions. Bars represent mean and standard deviation. No significant differences were found between patients and controls.


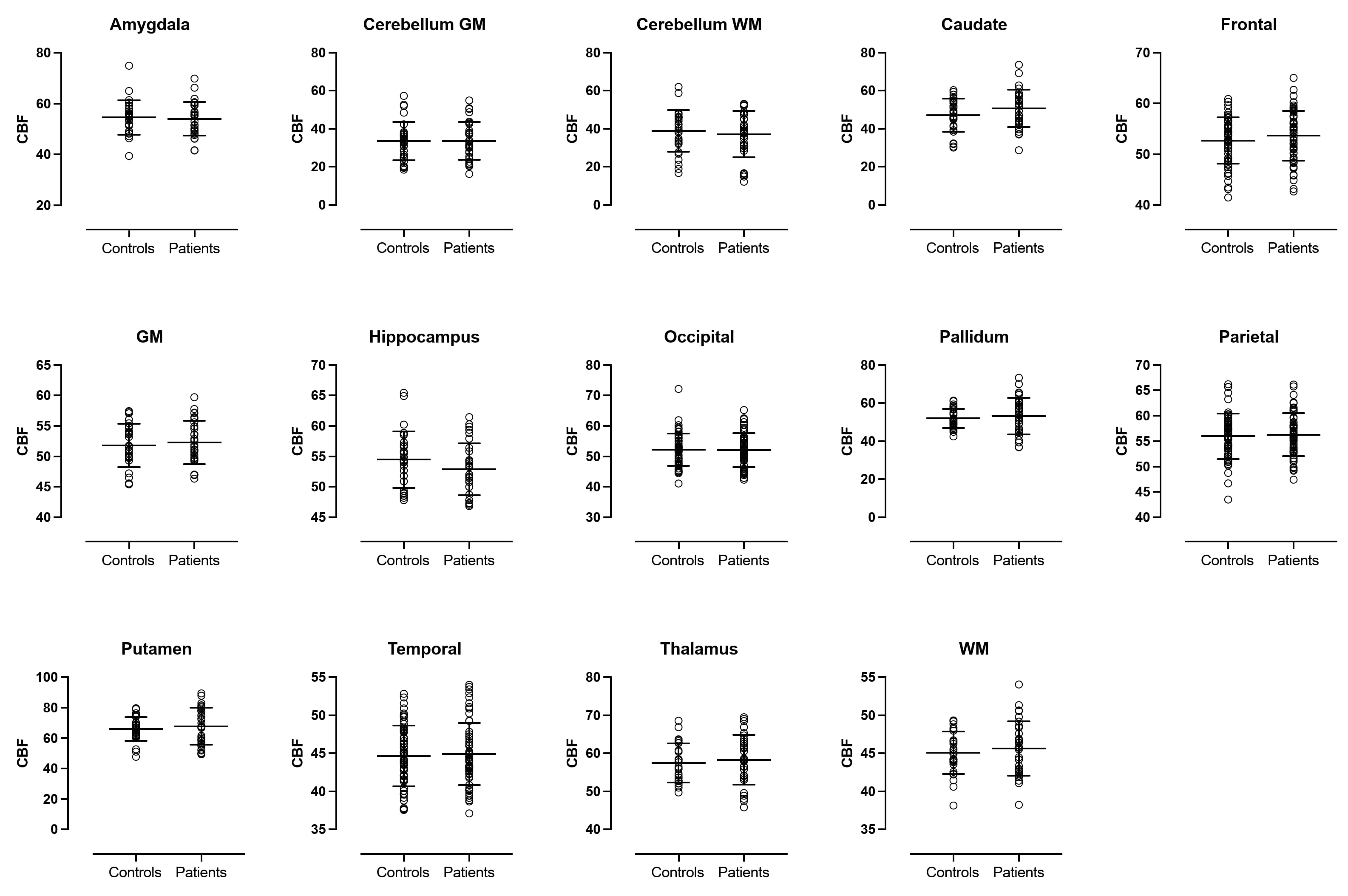


**Supplementary Figure 2:** Scatter plots for cerebral blood flow (CBF) in cortical- and subcortical regions. Bars represent mean and standard deviation. No significant differences were found between patients and controls.


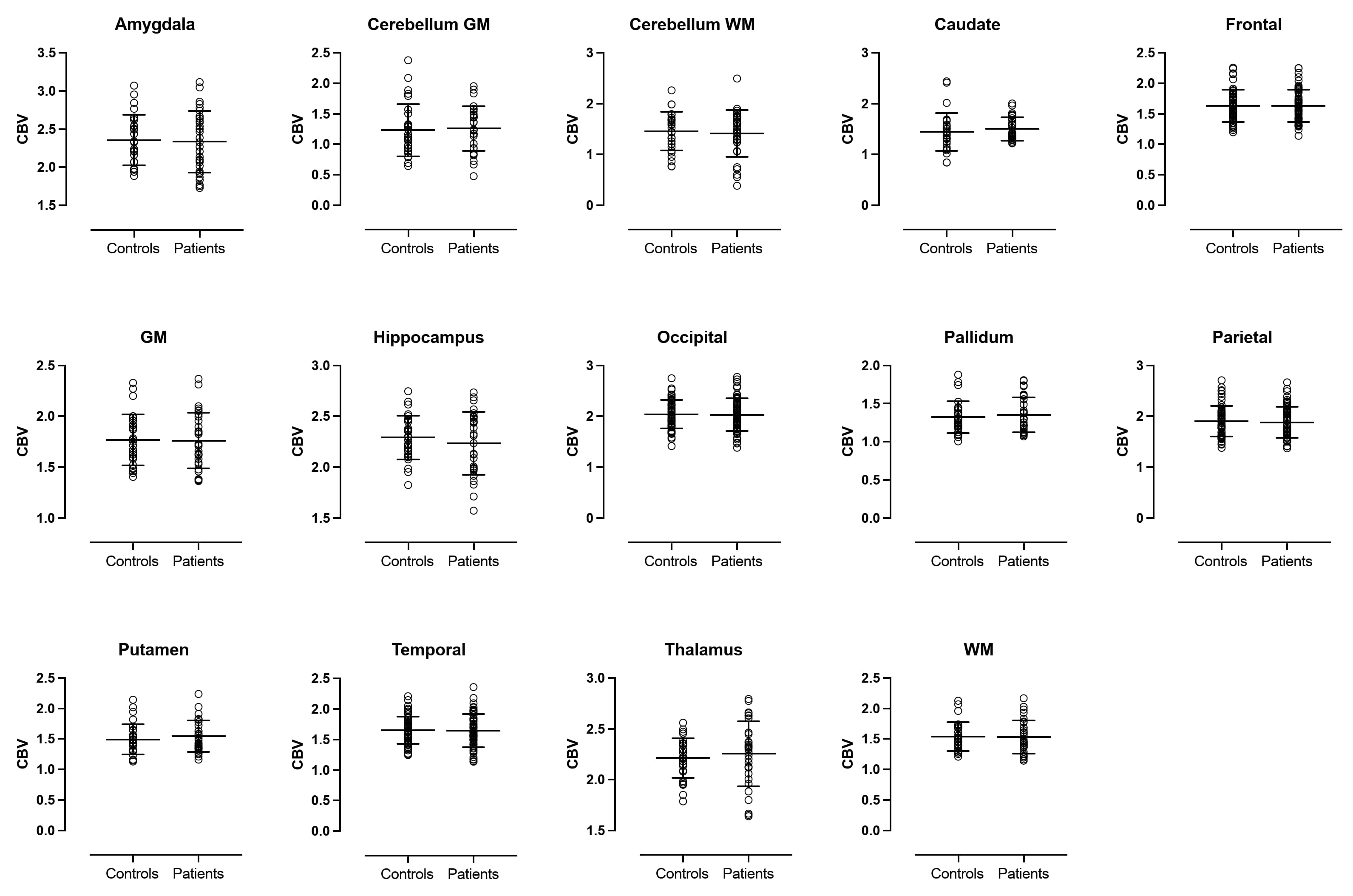


**Supplementary Figure 3:** Scatter plots for cerebral blood volume (CBV) in cortical- and subcortical regions. Bars represent mean and standard deviation. No significant differences were found between patients and controls.


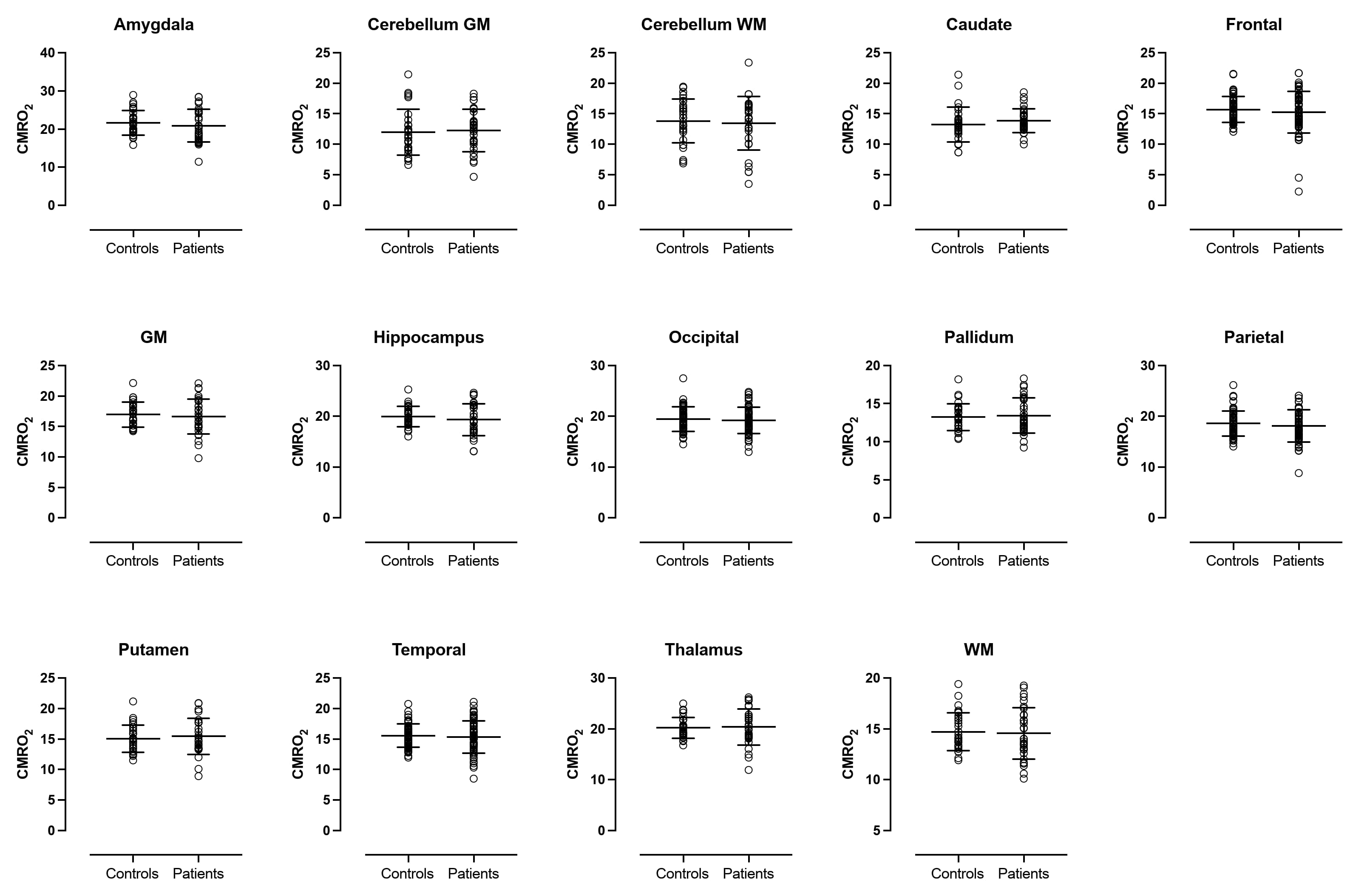


**Supplementary Figure 4:** Scatter plots for cerebral metabolic rate of oxygen (CMRO_2_) in cortical- and subcortical regions. Bars represent mean and standard deviation. No significant differences were found between patients and controls.


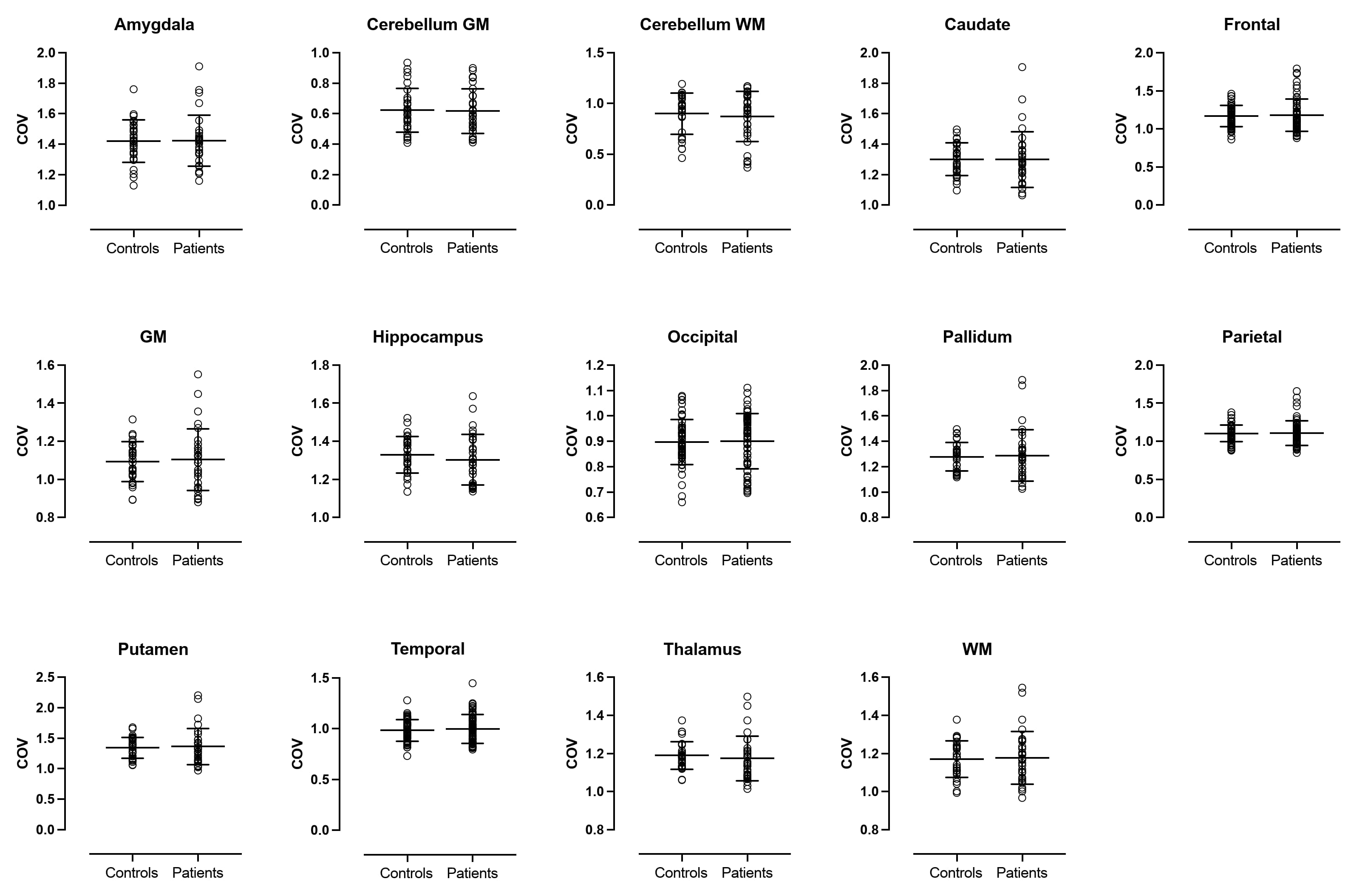


**Supplementary Figure 5:** Scatter plots for coefficient of variation (COV) in cortical- and subcortical regions. Bars represent mean and standard deviation. No significant differences were found between patients and controls.


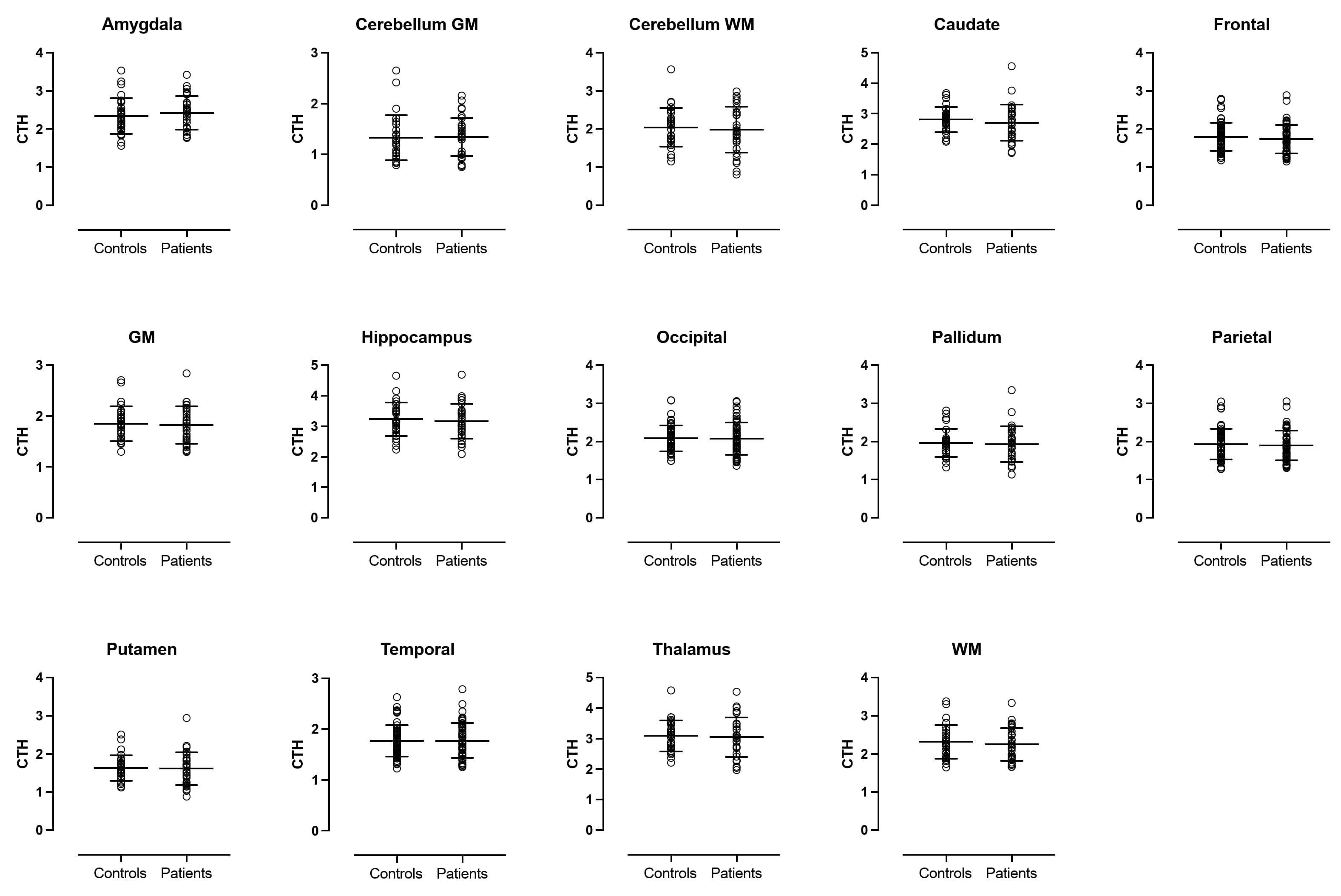


**Supplementary Figure 6:** Scatter plots for capillary transit time heterogeneity (CTH) in cortical- and subcortical regions. Bars represent mean and standard deviation. No significant differences were found between patients and controls.


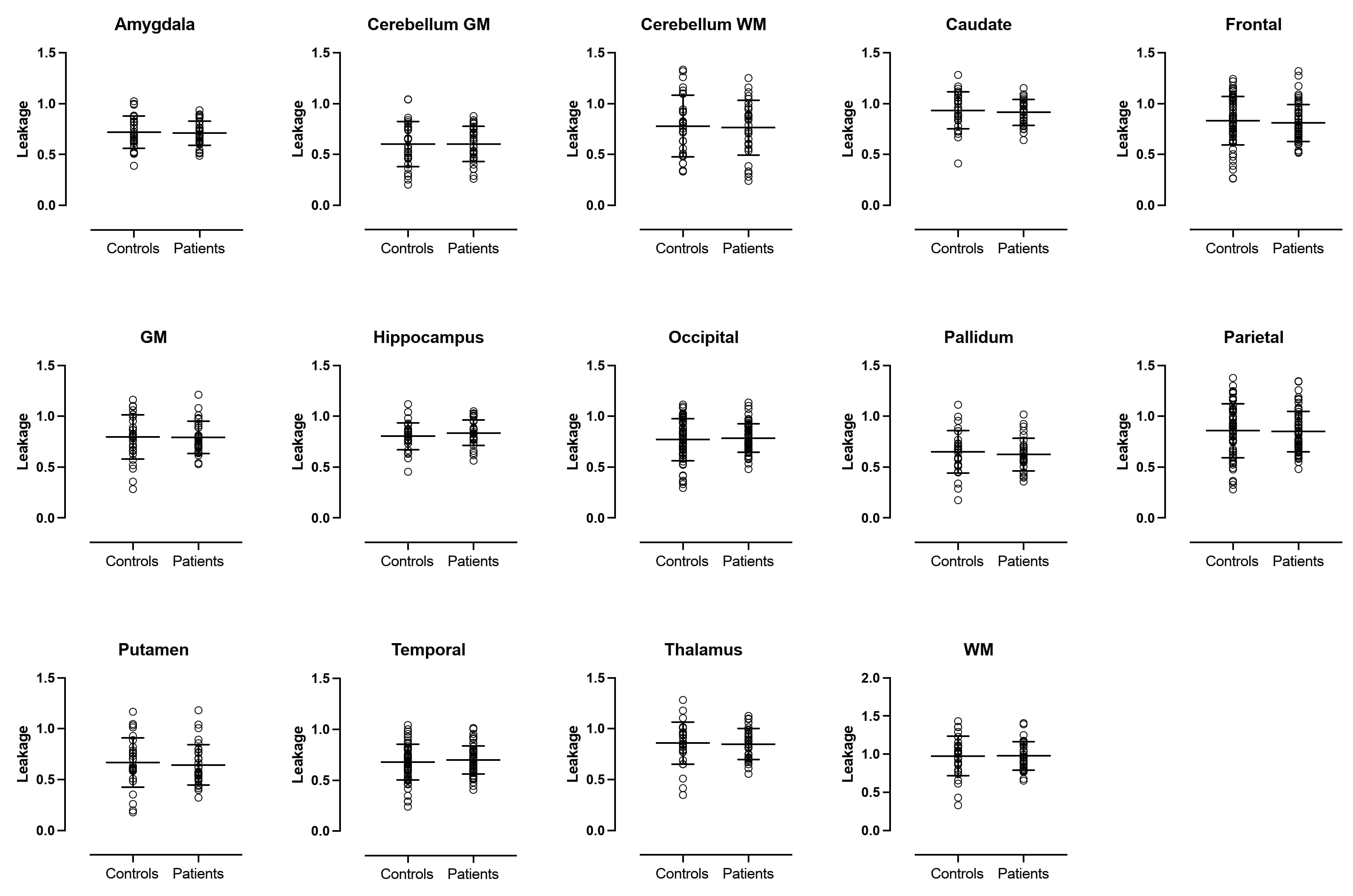


**Supplementary Figure 7:** Scatter plots for leakage in cortical- and subcortical regions. Bars represent mean and standard deviation. No significant differences were found between patients and controls.


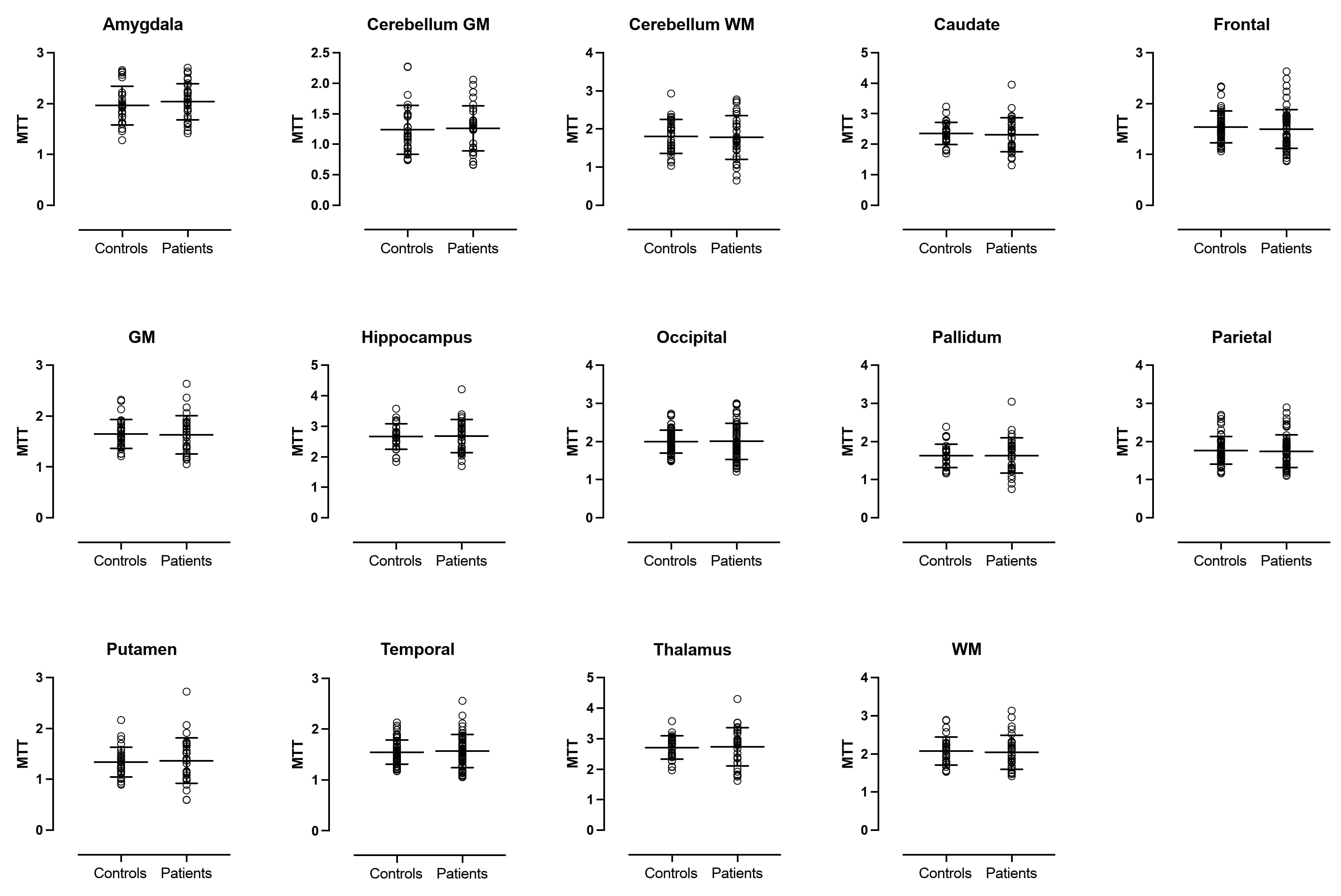


**Supplementary Figure 8** Scatter plots for mean transit time (MTT) in cortical- and subcortical regions. Bars represent mean and standard deviation. No significant differences were found between patients and controls.


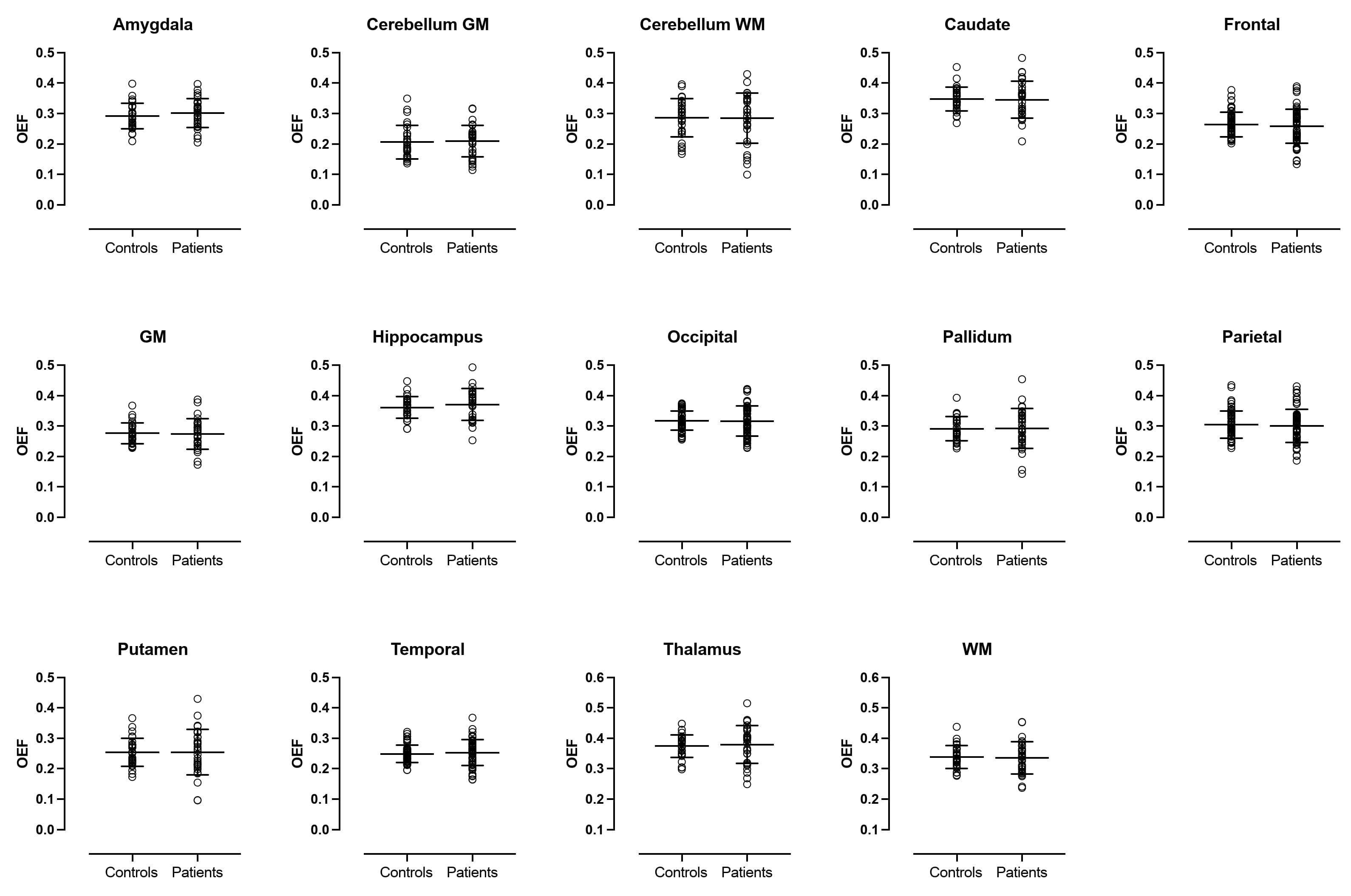


**Supplementary Figure 9:** Scatter plots for oxygen extraction fraction (OEF) in cortical- and subcortical regions. Bars represent mean and standard deviation. No significant differences were found between patients and controls.


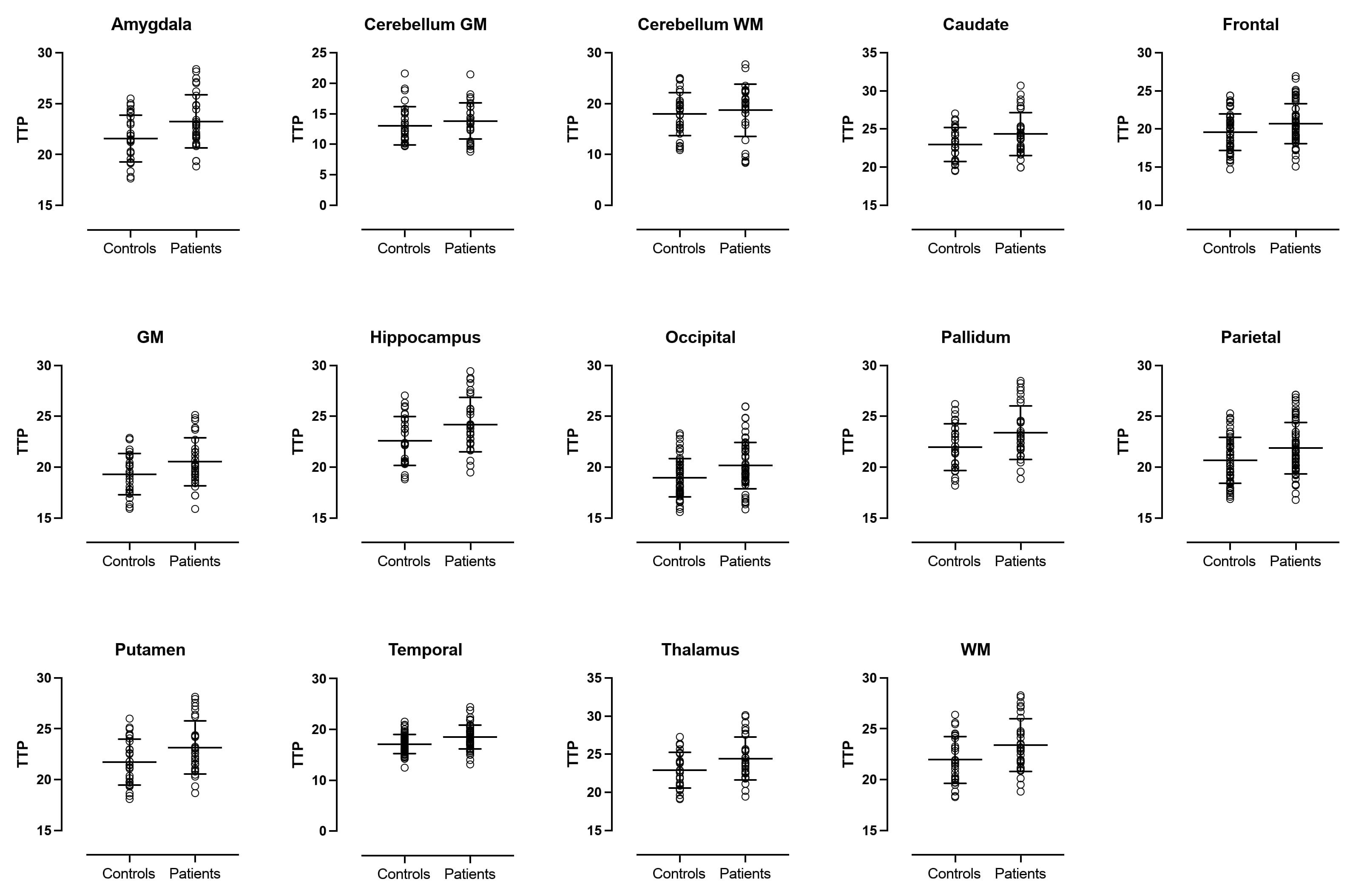


**Supplementary Figure 10:** Scatter plots for time to peak (TTP) in cortical- and subcortical regions. Bars represent mean and standard deviation. No significant differences were found between patients and controls.
